# AmyP53 Prevents the Formation of Neurotoxic β-Amyloid Oligomers through an Unprecedent Mechanism of Interaction with Gangliosides: Insights for Alzheimer’s Disease Therapy

**DOI:** 10.3390/ijms24021760

**Published:** 2023-01-16

**Authors:** Fodil Azzaz, Henri Chahinian, Nouara Yahi, Jacques Fantini, Coralie Di Scala

**Affiliations:** 1 INSERM UMR_S 1072, Aix Marseille University, 13015 Marseille, France; 2 Neuroscience Center—HiLIFE, Helsinki Institute of Life Science, University of Helsinki, 00014 Helsinki, Finland

**Keywords:** Alzheimer, Parkinson, lipid raft, ganglioside, oligomer, amyloid pore, calcium, AmyP53, therapy, adaptative peptide

## Abstract

A broad range of data identify Ca^2+^-permeable amyloid pores as the most neurotoxic species of Alzheimer’s β-amyloid peptide (Aβ_1–42_). Following the failures of clinical trials targeting amyloid plaques by immunotherapy, a consensus is gradually emerging to change the paradigm, the strategy, and the target to cure Alzheimer’s disease. In this context, the therapeutic peptide AmyP53 was designed to prevent amyloid pore formation driven by lipid raft microdomains of the plasma membrane. Here, we show that AmyP53 outcompetes Aβ_1–42_ binding to lipid rafts through a unique mode of interaction with gangliosides. Using a combination of cellular, physicochemical, and in silico approaches, we unraveled the mechanism of action of AmyP53 at the atomic, molecular, and cellular levels. Molecular dynamics simulations (MDS) indicated that AmyP53 rapidly adapts its conformation to gangliosides for an optimal interaction at the periphery of a lipid raft, where amyloid pore formation occurs. Hence, we define it as an adaptive peptide. Our results describe for the first time the kinetics of AmyP53 interaction with lipid raft gangliosides at the atomic level. Physicochemical studies and in silico simulations indicated that Aβ_1–42_ cannot interact with lipid rafts in presence of AmyP53. These data demonstrated that AmyP53 prevents amyloid pore formation and cellular Ca^2+^ entry by competitive inhibition of Aβ_1–42_ binding to lipid raft gangliosides. The molecular details of AmyP53 action revealed an unprecedent mechanism of interaction with lipid rafts, offering innovative therapeutic opportunities for lipid raft and ganglioside-associated diseases, including Alzheimer’s, Parkinson’s, and related proteinopathies.

## 1. Introduction

Neurodegenerative diseases, and in particular Alzheimer’s disease, are major public health scourges that will have a considerable impact on our health systems in the coming years [1]. About 50 million people are currently affected by Alzheimer’s disease worldwide, and according to the forecast, the number could reach more 100 million people in 2050 [1,2]. To date, there is no available solution to treat or prevent the disease, so that at best we can only try to relieve symptoms [3].

The paradigm that has dominated clinical research on Alzheimer’s disease for more than 30 years points to amyloid plaques as the root cause of the disease [4]. These plaques are essentially made up of the Alzheimer’s β-amyloid peptide (Aβ_1–42_), a small protein which self-aggregates in an aqueous medium to form larger assemblies by fibrillation [5,6,7]. Logically, most therapeutic strategies converged to target and destroy amyloid plaques, with the hope to cure the disease. Unfortunately, this approach has been unsuccessful because even if such treatments could actually decrease the burden of the amyloid plaques in the patients’ brains, they did not improve their cognitive status nor stop the evolution of the disease [8]. Thus, most immunotherapies with monoclonal antibodies targeting amyloid plaques have failed [9,10]. The only exception is Lecanemab, an antibody claimed to be more specific to Aβ protofibrils with limited cross-reaction with Aβ_1–42_ monomers or amyloid plaques [11]. Although the cognitive improvement associated with Lecanemab treatment is modest [12], this result has been interpreted as a confirmation that the molecular target Aβ_1–42_ is indeed the right one, but that the level of aggregation of the protein is the critical parameter controlling its neurotoxicity. A new concept is therefore emerging, according to which the most neurotoxic molecular species of Aβ_1–42_ corresponds to oligomeric forms and that it is these oligomers that should be targeted rather than the amyloid plaques [13]. In fact, this concept is not new. Since the early 1990s, an alternative theory has claimed that Aβ_1–42_ neurotoxicity is due to the protein’s ability to form small oligomers in the plasma membrane of brain cells [14]. These oligomers, referred to as amyloid pores, have a donut shape delimiting a central channel attracting the Ca^2+^ ions and allowing them to penetrate massively into the cells [15]. The entry of Ca^2+^ through amyloid pores triggers a neurotoxic cascade activating hyperphosphorylation of the Tau protein, oxidative stress, and functional dysregulation leading to neuronal death [13].

The role of membrane lipids (cholesterol, phospholipids, and gangliosides) in the process of oligomerization and formation of amyloid pores have been demonstrated by numerous studies [16,17,18,19,20,21,22,23,24]. Schematically, there are two ways by which oligomers can perforate brain cell membranes: (i) a punching hole mechanism of preformed Aβ oligomers, and (ii) the oligomerization of Aβ_1–42_ monomers subsequent to their insertion in the plasma membrane [4]. Both processes occur in lipid raft microdomains, involving gangliosides as the first attachment site for Aβ_1–42_ oligomers and monomers [25]. This strategic role of gangliosides is the Achille’s heel of Aβ_1–42_, as demonstrated by a broad range of experimental data. The toxicity of preformed oligomers can be prevented by the B subunit of cholera toxin, a classical GM1-binding protein [26]. Moreover, the assembly of Aβ oligomers into functional amyloid pores is blocked by metabolic inhibitors of ganglioside biosynthesis [27]. These data suggested that a lipid-raft-based therapy targeting brain gangliosides could represent a valuable alternative to anti-Aβ immunotherapies [28].

With the aim of developing new therapeutic options for Alzheimer’s disease patients, we decided to study the molecular mechanisms of Aβ_1–42_ binding to gangliosides. We identified a structurally related ganglioside binding domain in Aβ_1–42_ and in α-synuclein, the amyloid protein associated with Parkinson’s disease [29]. Then, we combined these two domains into a single designed chimeric α-synuclein/Aβ peptide that recapitulates the ganglioside-binding properties of its parental amyloid proteins [30]. This peptide, called AmyP53, has an exceptional safety and pharmacological profile, and it can be administered via the nose-to-brain route [31].

The mechanism of action of AmyP53 is clearly to restrict the accessibility of Aβ monomers and oligomers to gangliosides [25]. However, the molecular details of AmyP53 binding to gangliosides have not been totally elucidated. In the present study, we used various experimental approaches to study the competition between AmyP53 and Aβ_1–42_ monomers. At all scales (atomic, molecular, and cellular), AmyP53 outcompetes Aβ_1–42_ binding to ganglioside GM1, which is the main target of Aβ_1–42_ in lipid rafts of brain cells. In silico approaches in lipid raft model systems gave new insights in the molecular mechanism of action of AmyP53 and revealed an unprecedent mechanism of molecular interaction of a therapeutic peptide with cell surface gangliosides.

## 2. Results

### 2.1. Interaction of AmyP53 and Aβ_1–42_ with GM1 in Lipid Rafts

In a first series of experiments, we analyzed the behavior of the AmyP53 peptide in the presence of a reconstituted membrane system mimicking a lipid raft in its membrane environment. This system consists of a ternary monolayer containing the ganglioside GM1, cholesterol, and phosphatidylcholine (POPC). Thus, GM1 and cholesterol spontaneously cluster to form functional rafts separated by a matrix of POPCs. This membrane forms a monolayer at the water–air interface, in which the polar parts of the lipids are directed towards the aqueous phase and the apolar parts flush with the surface of the water in contact with the air.

**Figure 1:**
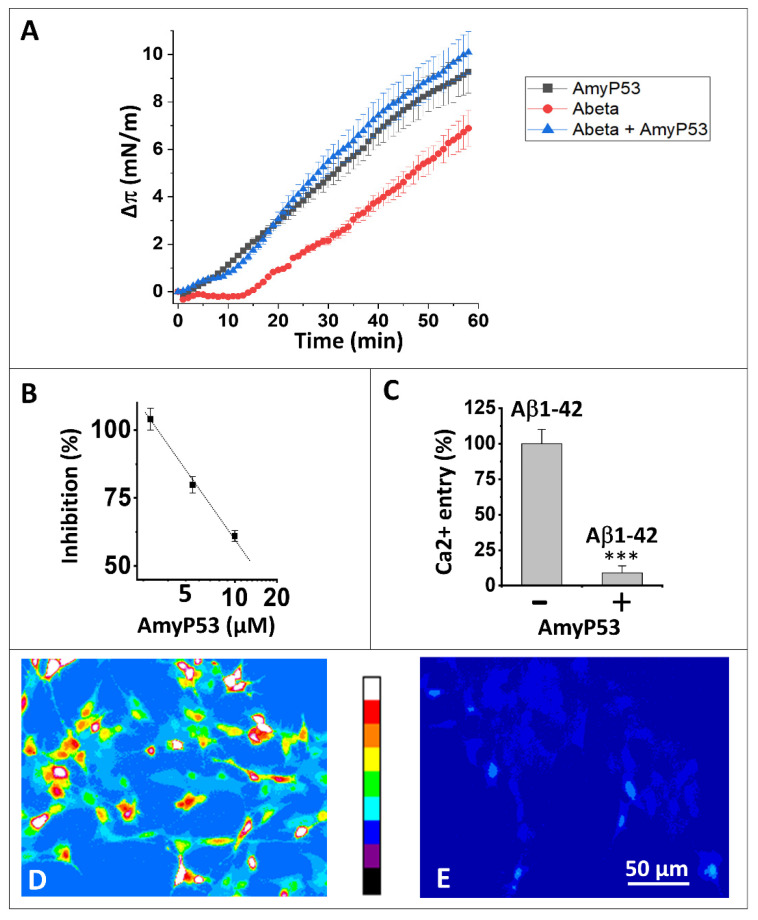
AmyP53 is a competitive inhibitor of Aβ_1–42_ binding to gangliosides and subsequent neurotoxicity events. (**A**) Effect of AmyP53 (10 µM), or Aβ_1–42_ (10 µM), or a mixture of both (each at 10 µM) added underneath a ternary monolayer (POPC: GM1: cholesterol, 10: 5: 5 mol: mol: mol). The data were expressed as the surface pressure increase (Δπ) ± SD (n = 3) over the time (left panel, 60 min; right panel, 20 min). (**B**) Inhibitory effect of AmyP53 for Aβ_1–42_ binding to aged SHSY-5Y cells. (**C**) Ca^2+^ fluxes induced by Aβ_1–42_ (220 nM) added for 1 h to aged SHSY-5Y cells preloaded with Fluo-4AM, in absence (−) or presence (+) of AmyP53 (220 nM). Data are expressed mean ± SD of 3 distinct experiments (Student’s test ***; *p* < 0.05). Corresponding micrographs taken at 60 min show the effect of Aβ_1–42_ alone (**D**) or Aβ_1–42_ incubated in presence of AmyP53 (**E**). The images in panels (**D**,**E**) show pseudocolor representations of cells at the end of the incubation (warmer colors corresponding to higher fluorescence, according to the scale between the micrographs).

The AmyP53 peptide is injected into the aqueous phase (Figure 1A). It immediately triggers an increase in surface pressure (∆π expressed in mN/m and recorded in real time with a computerized microtensiometer) corresponding to its insertion between the polar headgroups of gangliosides at the monolayer surface. The same experiment carried out with the amyloid protein Aβ_1–42_ also causes an increase in surface pressure, but with a lag time of about twenty minutes.

These results are in agreement with our previous experiments showing an interaction between Aβ_1–42_ and raft gangliosides. The delay observed in this experiment, compared to the results obtained in a monolayer system consisting exclusively of GM1-cholesterol rafts [32], indicates that the presence of the surrounding phospholipids delays the access of Aβ_1–42_ to GM1.

AmyP53 and Aβ_1–42_ present very different interaction profiles, so it was interesting to analyze the behavior of the two molecules in this membrane system (Figure 1A). It clearly appears that when these two molecules are incubated simultaneously with the GM1-Cholesterol-POPC ternary monolayer, the interaction profile corresponds completely to that of AmyP53, since the interaction starts without any delay. We conclude from this experiment that AmyP53 reaches its GM1 target faster than Aβ_1–42_.

### 2.2. AmyP53 Is a Competitive Inhibitor of Aβ_1–42_ Binding to Aged Neural Cells

In a second experiment, we analyzed the ability of AmyP53 to inhibit the binding of the amyloid protein Aβ_1–42_ to aged, GM1-positive SH-SY5Y nerve cells (Figure 1B). The results show a dose-dependent inhibition of AmyP53 on the cell binding of Aβ_1–42_ in agreement with the experiments of competitive kinetics. We can therefore extrapolate the behavior of AmyP53 observed in a membrane model system to the situation in the presence of living cells.

### 2.3. AmyP53 Blocks the Formation of Ca^2+^-Permeable Amyloid Pores

We then analyzed the functional consequences of this competitive inhibition. For this, we have developed a system of aged brain cells that are particularly sensitive to the neurotoxicity of amyloid proteins, such α-synuclein and Aβ_1–42_ [25,27]. These cells are first loaded with the indicator dye Fluo4-AM, the fluorescence of which is affected by the binding of Ca^2+^ ions [33]. Aged SH-SY5Y cells are then incubated in the presence of nanomolar concentrations of monomeric Aβ_1–42_ which inserts into the plasma membrane and self-assembles into amyloid pore-like oligomers permeable to Ca^2+^ ions.

As shown in Figure 1C, nanomolar concentrations of Aβ_1–42_ triggered an increase in intracellular Ca^2+^ due to the formation of amyloid pores in the plasma membrane of aged SH-SY5Y cells. In presence of equimolar concentrations of AmyP53 in competition, almost no amyloid pore formation was abrogated and no Ca^2+^ entry could be measured. These data are based on the quantitative analysis of Ca^2+^ imaging in aged SH-SY5Y cells treated with Aβ_1–42_ alone (Figure 1D) or Aβ_1–42_ in competition with AmyP53 (Figure 1E). 

### 2.4. Timeline of the Journey of AmyP53 on the Surface of a GM1 Lipid Raft

**Figure 2:**
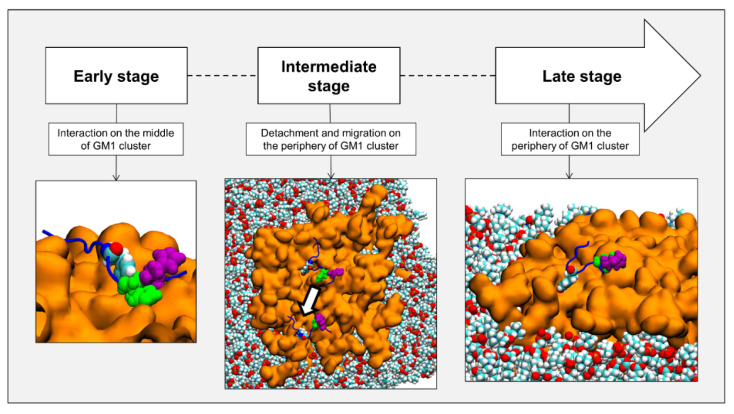
Chronologic events encountered by AmyP53 in our simulations. Gangliosides are depicted as orange surface, POPC phospholipids are depicted as spheres colored by atom name, and AmyP53 is represented as blue cartoon. Y6 is depicted as spheres colored by atom name, H9 is depicted as green spheres, and H10 is depicted as purple spheres.

Taken together, these data strongly suggest that AmyP53 binding to GM1 prevents the attachment of Aβ_1–42_ to lipid rafts. Hence, in presence of AmyP53, Aβ_1–42_ cannot interact with the plasma membrane of aged brain cells, explaining the absence of amyloid pores in AmyP53-treated cultures. However, the molecular details of AmyP53 binding to lipid rafts remained to be established. We used molecular dynamics simulations (MDS) to study the behavior of AmyP53 in the neighborhood of a lipid raft consisting of GM1 and cholesterol molecules at a ratio of 1:1 (mol:mol) surrounded by phospholipids. We initially placed AmyP53 above the lipid raft at a distance of 12 Å. After visualizing the trajectory, we identified three main events that we defined as early, intermediate, and late stages (Figure 2). The early stage consists of the first interaction of AmyP53 in the center of the lipid raft. This stage takes place between 0 and 30 ns. The second stage is the detachment and the migration of the peptide towards the periphery of the raft. This event starts at 30 ns and ends at 45 ns. Finally, the late stage is the stabilized binding of AmyP53 to the lipid raft.

### 2.5. Comparison of the Burial of Aromatic Residues of AmyP53 in the Lipid Raft Surface

**Figure 3:**
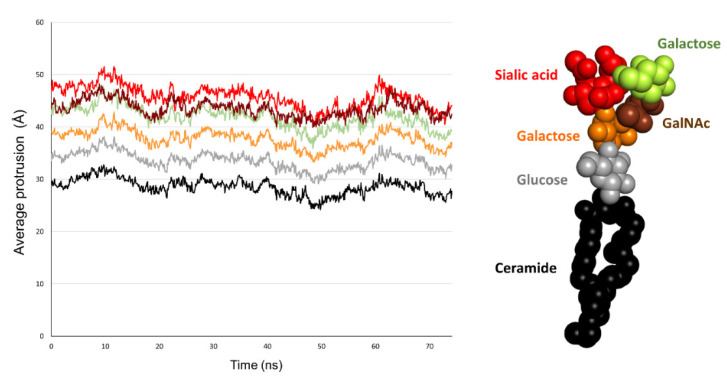
Plot showing the average of maximum value over the time along the z-axis for the ceramide part of GM1 molecules (black), glucose residues (grey), galactose residues (yellow), βGalNAc residues (brown), and sialic acids residues (red).

**Figure 4:**
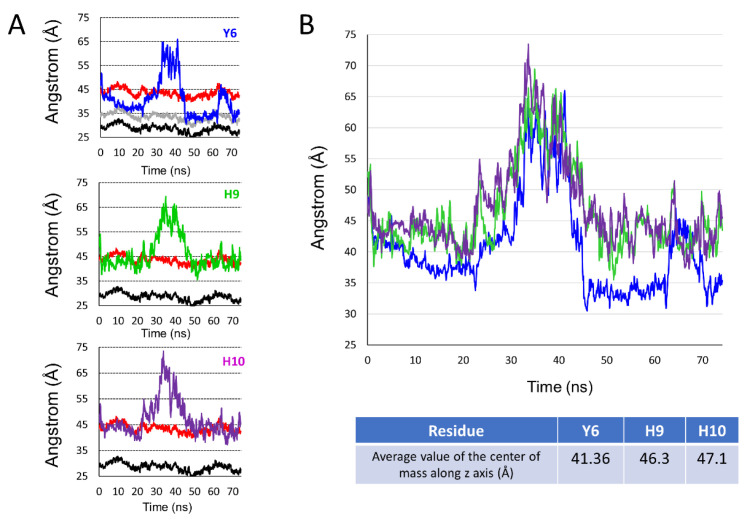
(**A**) Set of plots showing the center of mass over the time along the z-axis for Y6 (blue line, top plot), H9 (green line, mid plot), and H10 (purple line, bottom plot) compared to the average of maximum value over time along the z-axis for sialic acid (red line, all plots), glucose (gray line, top plot), and the ceramide part of GM1 molecules (black line, all plots). (**B**) Plot showing the center of mass along the z-axis over time for Y6 (blue line), H9 (green line), and H10 (purple line) and table below the plot showing the average value of the center of mass along the z-axis for each aromatic residue.

The fact that AmyP53 increased the surface pressure of a monolayer containing GM1 gangliosides (Figure 1A) indicates that the peptide dives into the membrane by inserting itself between the polar heads of the gangliosides. For this reason, we have developed a molecular modeling approach to measure the dip of the side chains of the peptide by taking as a benchmark the different structural parts of GM1. Among the amino acid residues involved in AmyP53-GM1 interactions, we identified the three amino acids with an aromatic structure: tyrosine-6 (Y6), histidine-9 (H9), and histidine-10 (H10). Thus, we investigated the burial degree of these aromatic residues at the surface of the lipid raft. For this purpose, we compared the value of the center of mass along the z-axis of Y6, H9, and H10 of AmyP53 with the value of the average protrusion of ceramide and sialic acids of GM1 molecules. The plots in Figure 3 show the average protrusion of each GM1 part over the time, taking the center of the lipid bilayer as reference. With no surprise, ceramide has the lowest value, meaning that it is the component that protrudes the least while the sialic acid has the highest value. To evaluate the burial level of Y6, H9, and H10, we measured the center of mass along the z-axis of each residue that we plotted with the average protrusion of sialic acid and ceramide (and glucose for Y6). Indeed, Y6 is the AmyP53 amino acid that is buried the most deeply on the surface of the GM1 cluster. One can observe that Y6 reaches the glucose residue of GM1 at the early and late stages of the simulation (Figure 4A, top) while H9 and H10 do not come deeper than the sialic acid residues (Figure 4A, mid and bottom plot). Then, we plotted for the three amino acid residues the value of the center of mass along the z-axis over the time and we calculated the average. As shown in Figure 4B, Y6 is the most buried residue with an average value of 41.36 Å, followed by H9 with 46.3 Å, and H10 with 47.1 Å. Additionally, it is worth noting that at the early stage (between 0 and 30 ns), Y6 slowly sinks into the raft, while at the late stage (between 45 ns and the end of the trajectory), Y6 dives quickly and deeper in the lipid raft.

**Figure 5:**
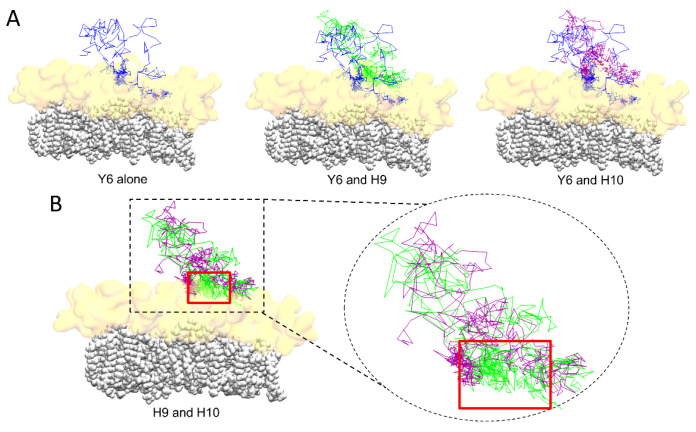
(**A**) Trail of aromatic residues of AmyP53 throughout the trajectory for Y6 alone (blue line, left), or compared to H9 (green line, middle), or compared to H10 (purple line, right). (**B**) Comparison of the trail of H9 (green line) and H10 (purple line). The ceramide chains of GM1 molecules are depicted as white spheres and the sugar moiety of GM1 is depicted as a transparent orange surface.

To compare more accurately the interaction of H9 and H10 with the lipid raft, we illustrated the travel trail throughout the trajectory of each aromatic residue, taking as reference their center of mass. The travel trail of Y6 is the one which penetrates the most deeply in the lipid raft (Figure 5A), consistent with the plots presented in Figure 4. The travel trails of H9 and H10 revealed that H9 is the one that establishes more interactions with the polar part of the lipid raft (Figure 5B). Overall, our computational data suggest that Y6 and H9 are the most critical amino acids that control the association of AmyP53 with the GM1 cluster.

### 2.6. Molecular Details of Raft-AmyP53 Interactions at the Early Stage

**Figure 6:**
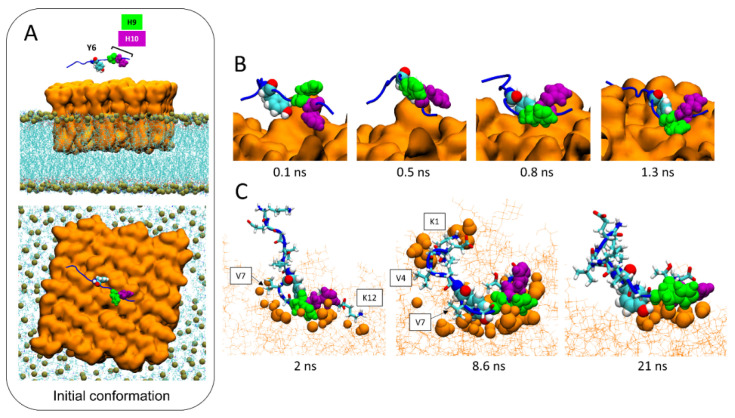
Molecular details of the interaction pathway of AmyP53 at the center of the lipid raft. (**A**) Initial conformation of AmyP53 above the sugar surface of GM1 cluster in front view (top snapshot) or top view (bottom snapshot). GM1 are represented as orange surface, phospholipids are represented as blue lines, and phosphorus atom of phospholipids are depicted as ochre spheres. (**B**) Molecular details of the reversal effect showing that H9 is favored over H10 to interact with the sugar surface of the lipid raft. AmyP53 is represented as blue cartoon in which Y6 is depicted as spheres colored by atom name, H9 is depicted as green spheres, and H10 is depicted as purple spheres. (**C**) Snapshots of three different conformations adopted by AmyP53 in the central area of the GM1 cluster. GM1 are depicted as thin orange lines, and atoms of GM1 that are at least at 3 Å of AmyP53 are represented as orange spheres. AmyP53 is represented as blue cartoon for which Y6 is depicted as spheres colored by atom name, H9 is depicted as green spheres, H10 is depicted as purple spheres, and all other residues are depicted as tubes colored by atom name.

In the initial conditions, AmyP53 is placed above the central zone of the lipid raft, with the side chain of its aromatic residues Y6 and H10 pointing towards the sugar moiety of GM1 molecules (Figure 6A). The dynamic of the peptide begins with a reversal effect which brings the side chain of H9 in contact with the lipid raft while the side chain of H10 points towards the opposite direction of the membrane (Figure 6B). However, H10 does not remain inactive. As shown by three different snapshots taken at 2, 8.6, and 21 ns (Figure 6C), we can see that H10 also participates in the interaction of AmyP53 with the raft through direct contacts with the sugar residues of GM1 molecules. At this stage, H9 and H10 resemble the open wings of a butterfly foraging on the lipid raft surface. In addition to these three residues (Y6, H9, and H10), we can also observe that lysine-1 (K1) and lysine-12 (K12) also interact with the lipid raft via hydrogen bonds and electrostatic interactions, whereas valine residues V4 and V7 interact with GM1 molecules via Van der Waals interactions.

### 2.7. Molecular Details of the Detachment and Migration of AmyP53 towards the Periphery of the GM1 Lipid Raft

**Figure 7:**
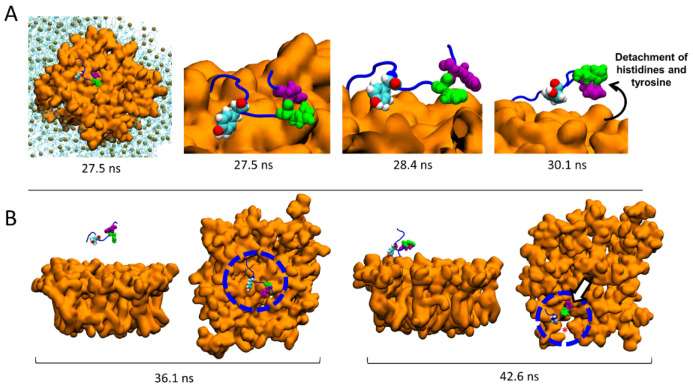
Migration of AmyP53 from the middle of the GM1 cluster to its periphery. (**A**) Molecular details of atomic events that triggered the detachment of AmyP53 from the middle of the GM1 cluster. (**B**) Set of snapshots showing the migration of AmyP53 towards the periphery of GM1 cluster in front and top views.

**Figure 8:**
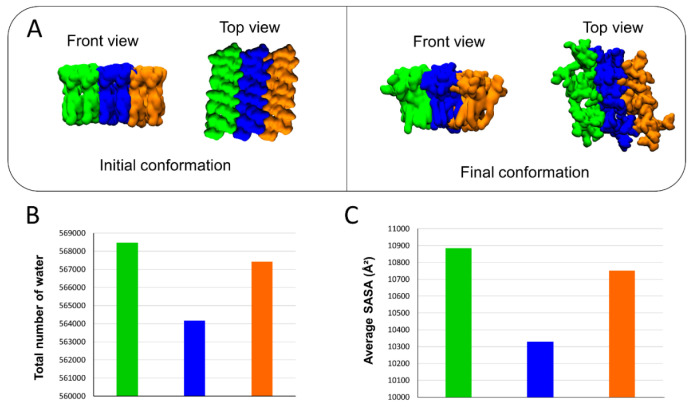
(**A**) Initial and final conformations of the GM1 lipid raft. The orange surface represents the periphery of the lipid raft that does not accommodate AmyP53. The blue surface is the middle part of the lipid raft, while the green color represents the surface which accommodates AmyP53. (**B**) Plot showing the total number of water molecules that surround the corresponding part of the lipid raft over time. (**C**) Average SASA (solvent-accessible surface area) for each different part of the lipid raft over the time.

The molecular details reveal that the detachment of AmyP53 from the surface of the lipid raft is triggered by a loss of affinity of aromatic residues for the central zone of the GM1 cluster (Figure 7A). Next, AmyP53 migrates to a crevice at the periphery of the lipid raft that appears to be intended to accommodate AmyP53 through a typical induced fit process (red asterisk in Figure 7B). After this observation, we studied how the polar regions of the raft adapt their structural dynamic in response to the presence of AmyP53 which clearly affects the conformation of GM1 sugars. For this, we have divided the GM1 cluster into three equal parts containing the same number of GM1 molecules (Figure 8). The orange part corresponds to the periphery of the lipid raft that does not accommodate Ampy53, the blue part represents the center of the GM1 cluster, and the green part the periphery of the raft that accommodates the peptide. If we compare the snapshots presenting the final conformation of the GM1 raft, we can observe that the green area is less packed than the orange one, whereas the blue zone is clearly the most packed (Figure 8A). To gain more accuracy in this observation we calculated the total number of water molecules interacting with each zone (with a cutoff of 2.5 Å) over the time, and then we calculated the average solvent-accessible surface area (SASA). The results presented in Figure 8B,C reveal that the central part of the lipid raft is the surface which is the least exposed to water molecules, indicating a strong packing of the polar moiety of GM1 gangliosides in this area. In contrast, the periphery of the raft which accommodates AmyP53 is the most exposed to solvent molecules.

### 2.8. Conformational Analysis of AmyP53 at the Periphery of the Lipid Raft

**Figure 9:**
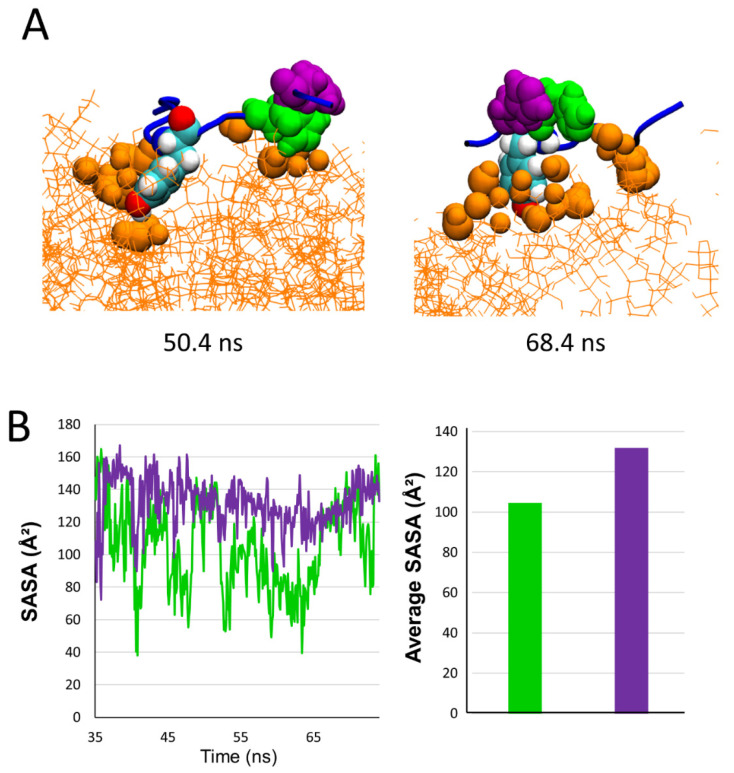
(**A**) Snapshot showing examples of conformations adopted by AmyP53 on the periphery of the lipid raft. GM1 are depicted as orange lines and GM1 atoms that are between 0 and 3 Å of protein are depicted as orange spheres. (**B**) Plot presenting the evolution of SASA over time for H9 (green line/histogram) and H10 (purple line/histogram) at the late stage, and histogram presenting the average SASA.

After migrating towards the periphery of the lipid raft, AmyP53 interacts with the polar part of GM1 mainly via its aromatic residues, among which Y6 is the most implicated (Figure 4 and Figure 5). In this raft area, we observed that H9 and H10 play a distinct role in the binding process of the AmyP53 peptide. The snapshots in Figure 9A show that H9 directly binds to the surface of the GM1 cluster while H10 interacts with H9 via π-π interactions. This effect could indirectly improve the binding of AmyP53 to the lipid raft by decreasing the solvent-exposed surface of H9, thereby stabilizing the interaction of H9 with the polar headgroup of GM1. To check this point, we plotted the SASA of H9 and H10 over the time at the late stage of the trajectory. The data revealed that the surface exposed to the solvent of H10 is greater than H9 with an average value of 132 against 105 (Figure 9B).

### 2.9. Competition between Aβ_1–42_ and AmyP53 for GM1 Binding

**Figure 10:**
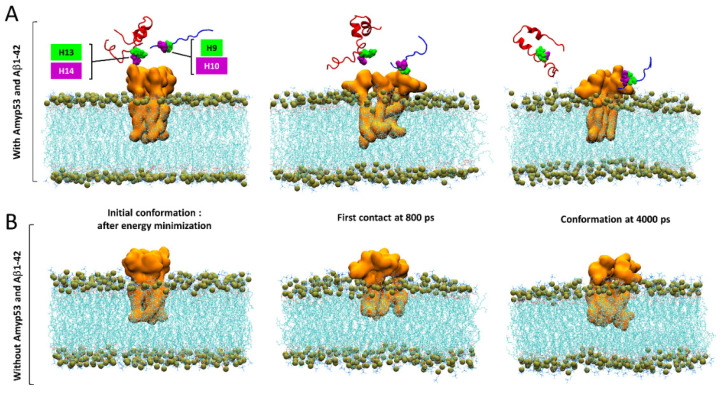
(**A**) Competition between AmyP53 and Aβ_1–42_ for interacting with GM1 molecules in front view. (**B**) Conformational landscape of GM1 molecules without AmyP53 and Aβ_1–42_. AmyP53 is depicted as blue cartoon while Aβ_1–42_ is depicted as red cartoon.

**Figure 11:**
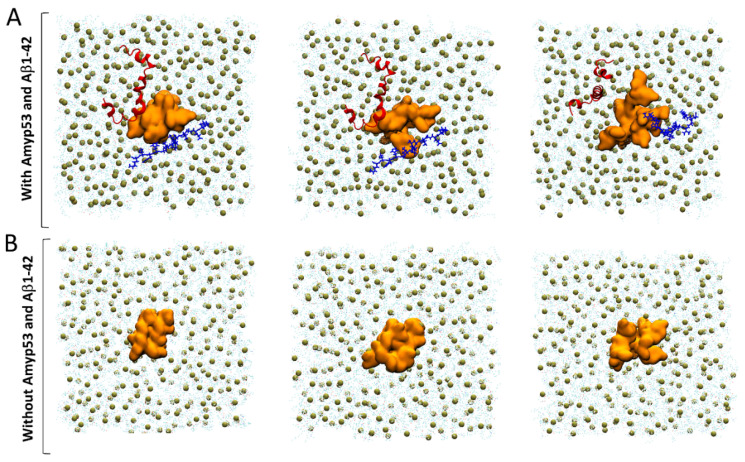
(**A**) Competition between AmyP53 and Aβ_1–42_ for interacting with GM1 molecules in top view. (**B**) Conformational landscape of GM1 molecules without AmyP53 and Aβ_1–42_. AmyP53 is depicted as blue cartoon while Aβ_1–42_ is depicted as red cartoon.

Finally, we used MDS to explain by which molecular mechanism AmyP53 outcompetes Aβ_1–42_ for interacting with GM1 gangliosides, as suggested by the data of Figure 1. To this end, we built a system consisting of four molecules of GM1 inserted in a POPC: cholesterol patch and we placed the two peptides above the polar headgroups of the gangliosides. Note that as showed by the snapshot in Figure 10A, the initial placement of Aβ_1–42_ is more favorable for the amyloid protein than the initial placement of AmyP53 to interact with GM1 molecules. Despite this handicap, AmyP53 is clearly the first molecule to enter in contact with gangliosides via its aromatic residue H9 (Figure 10A). The simulation reveals that Aβ_1–42_ is at a disadvantage to directly bind the sugar moiety of GM1 molecules because it undergoes much more intramolecular interactions than AmyP53. This typical U-folding of Aβ_1–42_ masks the aromatic residues that are critical for GM1 binding. As shown in Figure 10A, H13 and H14 are well exposed in the initial conformation, whereas they are engaged in a network of intramolecular interaction at 4000 ps. In contrast, the residues H9 and H10 of AmyP53 are, within this time range, less subjected to intramolecular interactions, allowing the therapeutic peptide to be more focused in establishing intermolecular interactions. In addition, we investigated if the simultaneous presence of Aβ_1–42_ and AmyP53 could influence the conformation of the sugar moiety of GM1 molecules. To this end, we built a system similar to the previous one, but we did not add Aβ_1–42_ and AmyP53. Although conformational differences are hardly visible in the front views (Figure 10A,B), the snapshots taken in the top views (Figure 11A,B) clearly show that the presence of Aβ_1–42_ and AmyP53 significantly affected the conformation of the polar part of gangliosides. Indeed, the sugar moieties of gangliosides are well packed together in the system without the protein/peptide duet (Figure 8), while they are more spaced apart in the system with both Aβ_1–42_ and AmyP53. Taken together, these observations suggest that the binding of AmyP53 to GM1 is not solely driven by the affinity and the capability of the peptide to penetrate the polar moiety of GM1, but rather through a fully cooperative mechanism in which the lipid raft plays an active role.

## 3. Discussion

In this study we analyzed the molecular mechanisms controlling the therapeutic effects of AmyP53, a peptide designed to prevent the neurotoxicity of amyloid proteins. This peptide was designed from strict specifications based on the identification of a common ganglioside-binding domain present on the Aβ and α-synuclein proteins, responsible, respectively, for Alzheimer’s and Parkinson’s diseases [30]. AmyP53 combines in the same molecule the ganglioside-binding properties of these two proteins. The fundamental characteristic of the two brain proteins from which AmyP53 is derived (Aβ and α-synuclein) is that they do not have a stable structure outside membrane environments. It is only by associating with raft gangliosides, which play the role of molecular chaperones, that these proteins structure themselves and acquire their neurotoxic potential [13,34]. Our previous work has elucidated the sequence of events transforming an inoffensive physiological protein into a highly neurotoxic molecular assembly that perforates the plasma membrane of brain cells and exposes them to a massive influx of Ca^2+^ ions [25,27]. These Ca^2+^ ions, which come from the extracellular space, trigger a neurotoxic cascade which activates all the hallmarks associated with Alzheimer’s disease and Parkinson’s disease: hyperphosphorylation of the Tau protein, microtubule disruption, oxidative stress, and neuronal loss [13,34].

These neurotoxic assemblies are oligomers that organize themselves into Ca^2+^-permeable pores (amyloid pores) in the plasma membrane of brain cells [35]. The sequence of events leading to the formation of amyloid pores begins with the attraction of amyloid proteins by a lipid raft [25]. This attraction involves electrostatic forces which position the areas of the protein with a positive surface electrostatic potential with the surface of the rafts, which is negatively charged due to the presence of gangliosides, such as GM1, within the rafts. If this step is blocked, the overall process of neurotoxicity of amyloid proteins is also blocked. The next step is the intervention of cholesterol from the rafts, which assists α-helical folding of the amyloid protein in the outer leaflet of the plasma membrane [36]. Finally, the cholesterol-assisted insertion and oligomerization process leads to the formation of the amyloid pore [18,20,36,37,38,39]. A similar mechanism allows preformed extracellular oligomers to insert themselves into the cell membrane after having been attracted by the gangliosides of the rafts, according to a perforation mechanism also dependent on the cholesterol of the rafts [36].

After elucidating these mechanisms, our team decided to propose a new therapeutic pathway targeting raft gangliosides [28]. It is in this context that the AmyP53 peptide was designed [30]. Like the proteins from which it is derived, which belong to the class of intrinsically disordered proteins (IDPs) [40,41,42,43], this peptide can adapt its conformation to gangliosides for an optimal interaction at the periphery of a lipid raft, where amyloid pore formation occurs. We have therefore eliminated solutions based on too rigid molecules (e.g., cyclic [44] or stappled peptides [45]) which would have had difficulty in adapting to the moving geometry of the rafts. In fact, we designed AmyP53 as a mini-IDP able to adapt its shape to the contours of the raft, hence the new concept of adaptive peptides.

The numerous results acquired with this adaptive peptide converge towards the notion that AmyP53 is a competitive inhibitor of the binding of amyloid proteins to a subset of raft gangliosides [13,34]. The new results presented here show that this property of AmyP53 is based on a kinetic advantage allowing AmyP53 to reach its target much faster than the Aβ_1–42_ protein. This is demonstrated by competition experiments in a membrane system mimicking a GM1 raft and its membrane environment consisting of phosphatidylcholine molecules. The particularity of this system is that Aβ_1–42_, which nevertheless has an excellent affinity for GM1 rafts [46], is not able to interact directly with this ternary membrane as it would with a binary GM1-cholesterol system [32]. We therefore took advantage of the delay in the interaction of Aβ_1–42_ with a ternary POPC-GM1-Cholesterol monolayer to find out whether AmyP53 also exhibited this lag of about twenty minutes in the interaction with this membrane. The results in Figure 1A show that this is absolutely not the case, since AmyP53 interacts immediately with this complex membrane. Furthermore, when the AmyP53/Aβ_1–42_ duet is incubated simultaneously with this type of membrane, the recorded response is clearly due to the interaction of AmyP53, which demonstrates the kinetic advantage of the therapeutic peptide over the amyloid protein. By binding more rapidly to raft gangliosides, AmyP53 inhibits the binding of Aβ_1–42_ to the plasma membrane of nerve cells (Figure 1B), which protects them from the massive influx of Ca^2+^ ions subsequent to the formation of amyloid pores (Figure 1C–E). It is important to note that the cellular system which allows the formation and detection of amyloid pores is a model of aged cells, used between the 20th and the 25th passage in culture. Cells freshly thawed after storage in liquid nitrogen are not permissive for the formation of amyloid pores, probably due to changes in the lipid composition upon membrane aging [47,48,49,50]. Such phenomena have been reported for several cell types used as models for studies of pathological processes [51,52,53].

Molecular dynamics simulations (MDS) allowed us to analyze, at the atomic level, the mechanisms controlling the interaction of the AmyP53 peptide with a GM1 ganglioside raft. These studies revealed previously undescribed mechanisms for a peptide–ganglioside interaction. The first surprise was to observe that the AmyP53 peptide is initially attracted to the central zone of the raft to which it binds almost instantaneously. This is probably due to the higher density of negative electric charges in the center of the raft, where the gangliosides are the most condensed. However, this initial interaction is not stable, and the peptide quickly detaches from this zone. This is the other side of the coin: the more the gangliosides are condensed, the less they are free to adjust their shape to stabilize the attachment of the AmyP53 peptide, despite a strong electrical field. Indeed, our study revealed that central gangliosides are less hydrated and less exposed to the solvent than peripheral ones (Figure 8) and are thus more packed. One should also note that the repulsive forces, due to the negative charge of GM1, are counterbalanced by the N-acetyl group of GalNAc which neutralizes the negative charge of the sialic acid. This effect, that we coined “NH trick”, is key to understanding the different topologies adopted by gangliosides (chalice-like at the edge of lipid rafts, condensed clusters in central areas) and their impact on protein binding [54]. Overall, this explains why the peptide detaches from the central zone of the raft, to reach the more mobile gangliosides of the periphery of the raft. This second attachment is much stronger than the first, the peptide gradually strengthening its anchoring to the surface of the raft. We have therefore analyzed three successive stages (early, intermediate, and late) describing the entire process. 

The simulations also made it possible to better understand the respective roles of the two histidine residues incorporated into the AmyP53 peptide sequence during its design. These two histidines are derived from the ganglioside-binding domain of Aβ_1–42_ [30]. It is a significant improvement that gives the peptide the ability to bind to a broader repertoire of gangliosides in the brain, and in particular GM1. However, our alanine scanning studies had curiously revealed that the ganglioside-binding motif of Aβ_1–42_ lost all GM1 recognition function if these two histidines were substituted, but unexpectedly also when each histidine was independently replaced by alanine [29,55]. Thanks to our new simulations, we were able to solve this enigma. In fact, it is the H9 residue that is most critical for GM1 binding. However, the H10 residue reinforces this interaction by coming into contact with H9, which drives out water molecules and gives an entropic contribution to the binding of H9. Moreover, as remarkably anticipated in a previous molecular modeling study performed with a simpler system [30], these two histidines adopt an open butterfly wing structure, which balances the peptide when it lands on the surface of the GM1 gangliosides (Figure 10). In this conformation, the positive electrostatic potential is harmoniously distributed over the surface of AmyP53, which is then strongly attracted by the negative electric field of the gangliosides of the raft. Finally, our data show that the central tyrosine residue of AmyP53 plays a key role in the insertion of the peptide between the polar head groups of gangliosides, confirming previous data obtained with the parental ganglioside-binding domains of Aβ_1–42_ and α-synuclein [29,30,55]. 

The second surprise of this study was to reveal the active role of raft gangliosides, whose conformational reorganizations (formation of crevices) seem to precede the binding of the AmyP53 peptide. We thus discovered that the attachment of AmyP53 to rafts is a dynamic process involving simultaneously several gangliosides and the peptide itself. Subjected to the concomitant influences of water molecules and gangliosides, AmyP53 is taken up by the raft extremely quickly and efficiently. Even in the presence of the amyloid protein Aβ_1–42_, initially positioned advantageously on the surface of the raft, AmyP53 wins the competition (Figure 10 and Figure 11). The kinetic advantage of AmyP53 is therefore well demonstrated at the different scales tested: atomic, molecular, physicochemical, and cellular. This advantage is also preserved on hippocampal slices incubated in the presence of Aβ_1–42_. In this case, AmyP53 prevents the shut-down of the electrophysiological activity of the neural network caused by Aβ_1–42_ [27].

Taken together, our results show that AmyP53 outcompetes Aβ_1–42_ binding to lipid rafts through an unprecedent mechanism of molecular interaction with gangliosides. AmyP53 is a first-in-class therapeutic molecule which inaugurates the category of adaptive peptides. This notion of adaptative peptides characterized by a certain conformational flexibility is not incompatible with target specificity. Indeed, the sequence selected for the design of AmyP53 is derived from a specific ganglioside-binding domain, a functional property that was intentionally, rationally, and specifically improved [30]. This augmented ganglioside-binding activity makes AmyP53 a promising solution that will be administered by the nose-to-brain route [31] and tested clinically for patients suffering from Alzheimer’s or Parkinson’s diseases. Such lipid-raft-based therapies could prove to be particularly appropriate for the treatment of diseases caused by a mechanism controlled by membrane gangliosides. In this respect, AmyP53 is the first therapeutic molecule specifically designed to target brain cells gangliosides [31,56].

## 4. Materials and Methods

### 4.1. Materials 

The Aβ_1–42_ was purchased from rPeptide (Watkinsville, GA, USA), dissolved in 1% NH_4_OH at a concentration of 1 mM, and frozen at −20 °C in working aliquots. The AmyP53 peptide was obtained from Schafer-N (København, Denmark). All peptides and proteins have a purity > 95% as assessed by HPLC. All lipids were purchased from Matreya (State College, PA, USA). Ultrapure apyrogenic water was from Biorad (Manes La Coquette, France). SH-SY5Y cells were from ATCC (Manassas, VA, USA) and Fluo-4AM was from Invitrogen (Waltham, MA, USA).

### 4.2. Monolayers Studies

The interaction of Aβ_1–42_ and AmyP53 with reconstituted lipid raft membranes was studied with the Langmuir film balance technique [57] using a Kibron Inc. (Helsinki, Finland) microtensiometer as previously described [29,55]. Lipid mixtures (GM1, cholesterol and phosphatidylcholine) were spread on pure water and allowed to equilibrate for 5 min. The peptide (or the protein, or both) was injected in the aqueous subphase with a 10 µL Hamilton syringe, and the surface pressure increases (Δ*π*) were continuously recorded as a function of time. The data were analyzed with the FilmWareX program (Kibron Inc.).

### 4.3. Cell Culture

SH-SY5Y cells (American Type Culture Collection) were grown in Dulbecco’s Modified Eagle Medium Nutrient Mixture F12 (DMEM/F12) with 10% fetal calf serum, glutamine (2 mM), and penicillin (50 U/mL)/streptomycin (50 µg/mL), and maintained at 37 °C with 5% CO2 as previously described [25]. Aged cells were used between passage 20 and passage 25.

### 4.4. Aβ_1–42_ Binding to SH-SY5Y Cells

The binding of biotin-labeled Aβ_1–42_ [58] to aged SH-SY5Y cells was performed as described previously [30]. Briefly, aged SH-SY5Y cells were seeded in 96-well plates at a density of 30.000 cells per well. After three days, the cells were rinsed in phosphate-buffered saline (PBS) and fixed with paraformaldehyde. After rinsing in PBS, the cells were incubated for 1 h with lipid-free bovine serum albumin (2%), and then with biotin-labeled Aβ_1–42_ for 2 h at a concentration of 8 µg.mL^−1^, in absence or presence of AmyP53 at the indicated concentrations. The cells were then rinsed and incubated with Streptavidin-HRP (Sigma, St Louis, MO, USA) for 1 hr. Sigma-Fast OPD (Sigma) was used as a revealing agent. The reaction was stopped with H_2_SO_4_ 2N and the absorbance was measured at 492 nm. Specific Aβ_1–42_ binding was estimated against blank experiments.

### 4.5. Ca^2+^ Flux Experiments

Intracellular Ca^2+^ levels were measured with the Ca^2+^-sensitive dye Fluo-4AM (5 µM) as described previously [25,27,56]. For comparative studies (absence or presence of AmyP53 at a concentration of 220 nM), the value obtained with cells treated with 220 nM Aβ_1–42_ alone was considered as 100%. All experiments were performed at 30 °C during 1 h.

### 4.6. Statistical Analysis

The statistical significance of the data was evaluated with the Student’s test.

### 4.7. Molecular Modeling

The therapeutic peptide AmyP53 [31] was modelized de novo using HyperChem [59]. The Charmm topology and parameters of AmyP53 were generated using the tool “Automatic PSF Builder” on the software VMD [60]. The GM1 lipid raft was modelized on VMD and the initial coordinates and the topology of cholesterol and GM1 were obtained using the tool “Bilayer Builder” on Charmm-GUI [61,62]. The lipid raft was inserted into a POPC bilayer obtained from Charmm-GUI and AmyP53 was placed above the central area of the lipid raft. The environment was solubilized and neutralized with Na^+^ and Cl^-^ counter ions at a final concentration of 0.15 mol/L using the tools “Add solvation box” and “Add ions” in VMD. The systems were simulated using the software NAMD 2.14 for Windows 10 coupled with the force field CHARMM36m [62,63,64]. The systems were minimized (10,000 steps) to remove all bad contacts between atoms and equilibrated at constant temperature (310 K) and constant pressure (1 atm). Then, productions runs were performed. The cutoff for the calculation of non-covalent interaction was set at 12 Å and the PME algorithm was used for the calculation of long-range electrostatic interaction in a periodic system.

## Data Availability

Not applicable.
